# Some Neuroses of the War

**Published:** 1916-07

**Authors:** J. Michell Clarke

**Affiliations:** Professor of Medicine, University of Bristol; Lieut.-Col., R.A.M.C.(T.)


### SOME NEUROSES OF THE WAR.

BY

J. MICHELL CLARKE, M.A., M.D., LL.D., F.R.C.P.,

*Professor of Medicine, University of Bristol; Lieut.-Col., R.A.M.C.(T.).*

THE following observations are based on cases seen in the Neurological Department of the 2nd Southern General Hospital. I propose to deal with the clinical aspect of the various neuroses met with amongst soldiers in this war. The majority are due to more than one factor. Some of the causes are psychical—terror, noise, the horrifying sights and experiences of the trenches—others physical; apart from actual wounds, the concussion of the air due to high explosives, burial in a trench, respiration of noxious gases, especially CO, as Dr. Mott has shown, more or fewer of which have been at work in individual cases of the condition known as shell-shock.

In many of the affections here considered it might justly be asked in what sense the term neurosis is used. According to Gould, a neurosis is an abnormal nervous action or an affection of the nerves or nerve-centres of a functional nature. I think we shall be on firm ground for clinical purposes if we exclude all cases which present any one or more of the definite clinical signs which we have learnt to associate with structural change in the central nervous system. At any rate, if we take this as a working hypothesis, we shall not go far astray. That the present conceptions of what constitutes a functional, as apart from an organic lesion, may require modification is quite likely, and only to be expected from the lines on which neurological investigation has hitherto proceeded. The distinction cannot be based upon the presence or absence of an external lesion, for it has been shown by Gordon Holmes, Mott and others that the effect of high explosives may be to produce organic lesions of the central nervous system, sometimes intense and localised, at others wide-spread and of slight intensity, in the absence of any sign of external injury. But in such cases, always in the former and generally in the latter, there will be clinically the accepted signs of organic disease, *i.e.* of structural changes. It seems to me, however, possible that minute multiple lesions of the central nervous system, especially if wide-spread, may, though the damage from any one of them is negligible, through a massed effect give rise to symptoms or signs not recognisable by present clinical methods of investigation as due to an organic lesion, but rather to those of functional disturbance or neurosis.

Further, that there would be all grades of such affections from what seemed to be a pure neurosis at one end leading through more doubtful cases to those at the other end, with undoubted signs of structural changes.

The neuroses of war will be partly due to the same causes as in civil life, and partly to other and special causes. It is to be expected, therefore, that we should meet with the familiar neuroses of civil practice, and also with other conditions presenting unfamiliar or special features. This may be made the basis of a preliminary classification.

Amongst the former will be easily separated the cases of hysteria, because the signs of this particular neurosis are well known and easy of recognition. Most of them are quickly cured by accepted methods when their nature is recognised. Cases of recent origin are more amenable to treatment than those of long standing, hence one importance of prompt recognition. If we take these cases first, we find that they comprise the ordinary manifestations of hysteria, viz. monoplegias, paraplegias and hemiplegias, with or sometimes without the pathognomonic sensory disorders and muscular contractures, affections of the special senses, such as deafness or amaurosis, and of special nervous mechanisms such as speech, and of anorexia or vomiting.

When hysterical paralysis of one limb is caused by a wound, the wound may be superficial or deep, slight or severe, the paralysis may come on at once after the injury, or not for some considerable time. The position of the wound is sometimes an aid to diagnosis, for it may be so situated that it could not by injury to nerves produce the paralysis present. Most commonly the paralysed parts are distal to the injury, or do not extend farther centrally than the position of the wound. There is generally anæsthesia of the well-known hysterical type, that is to say, covering an area like a glove or sleeve in the upper and like a stocking or sock in the lower limb or the whole limb may be anæsthetic as far as the shoulder or the groin. The upper limit of the anæsthesia is transverse to the long axis of the limb, as a rule is sharply defined, and the boundaries of the loss of the different forms of sensation are coterminous. All forms of sensation may be lost together, but those to light touch and pain (pin-prick), are more frequently affected than to heat or cold. Sensation to either hot or cold may be preserved and the other lost, or cold felt as warm. So that certain forms of dissociation of sensation are sometimes met with, and these anomalies may vary over different parts of the anæsthetic area. Attention to the distribution and characters of the anæsthesia rarely leaves any doubt as to its true nature. The affected limb is often cold, bluish-red, and sometimes slightly adematous, (œdeme bleu of Charcot).

The above points may be illustrated by two cases. In the first there was a bullet wound of the forearm just below the elbow ; paralysis of all movements of the forearm and hand came on shortly after the injury. When examined six weeks later there was general loss of movement of forearm and hand, without muscular wasting or alteration of electrical reactions. The hand was blue and cold. There was " long glove anæsthesia," its upper limit ending sharply at the level of the wound. Under treatment recovery was complete in a fortnight.

The second case was wounded slightly in the shoulder on 12th March. There was no paralysis at first, but in May he began to lose power in the hand, and this gradually increased until the hand became completely paralysed. He did not come under my observation until December, when he had had complete paralysis of the hand, of the long flexors and extensors of the fingers, as well as of the small muscles, for some months. Efforts to move the hand resulted in violent contraction of the arm muscles. There was no wasting during this long time, and electrical reactions were normal. Over the hand and to about four inches above the wrist there was complete loss of all forms of sensation, including those of pressure, sense of position and movement, and cold, however, was felt as warm. He also made a rapid and complete recovery.

The cause need not be a wound. Just as occurs in civil practice, any injury may produce hysterical monoplegia. Thus a man came under treatment for paralysis of the leg and foot after a Pott's fracture. There was " stocking anæsthesia " except on the sole of the foot, which was hyper-æsthetic. In other cases the cause appeared to be exposure to cold and wet. One patient was at first paraplegic, then the left leg recovered, leaving the right paralysed. This illustrates another feature of some of these cases in which an originally extensive paralysis leaves a more limited one as a residuum. Although he alleged complete inability to move the right leg, yet when it was picked up he held it extended some distance above the bed for some minutes before letting it drop. There was anæsthesia of the foot and leg up to the knee. He was got out of bed, shown various movements of the leg, encouraged to walk, at first with support, then without it, and was discharged fit for duty in three weeks.

In another case the patient was similarly at first paraplegic after " frost-bite " : the right leg soon recovered, but the left remained paralysed. In this instance, besides the loss of power, there was contraction of the extensors of the foot, keeping the foot strongly dorsi-flexed. Anæsthesia to touch, pain, sense of position and movement extended as high as the knee and was complete, so that without looking at his foot he had no idea of its position. He also made a rapid recovery.

Hysterical contractures of the limbs without paralysis are not so common, and I have met with no examples of a pure contracture so far in these war neuroses.

In long-standing hysterical contracture sensory disorders are often absent, and less often they are absent in cases of paralysis, and the diagnosis without this aid is certainly more difficult. Hysterical paralysis and anæsthesia may complicate the paralysis due to an organic lesion, when the functional disturbance has to be unravelled from that directly due to the lesion. With a little care the distinction can generally be made. Lapse of time is often an aid, as the functional disorder tends to clear up or to alter whilst the case is being investigated and treated. Sometimes hysterical disorders of motion—especially perhaps contracture—or of sensation are grafted on to a lesion of a peripheral nerve or nerves, but the commonest complication of this kind is for a hemi-anæsthesia of the whole body to accompany a lesion of the cerebrum. Sometimes there is hemi-paresis as well, but one must bear in mind that if hysterical anæsthesia is profound, and affects deep sensation, this generally involves loss or impairment of movement of the affected limbs.

In one case of wound of the back there was a complication of a mild Cauda Equina lesion with hysterical paresis of the legs. A bullet was removed lying external to the spinal column at the level of upper part fourth lumbar vertebra; there was a limited lesion of the Cauda Equina. At first he could not empty his bladder, and had some loss of control over the rectal sphincter. Some time after the injury paresis and numbness of the legs came on gradually. When seen six months after it the bladder and rectal symptoms had cleared up, but on the right buttock there was a well-defined area of loss of all forms of sensation in the field of the fourth sacral root; on the left side it was more extensive, involving as well part of the field of the third sacral. On both sides the sensory loss was of the peripheral type, the epicritic being slightly larger than the protopathic loss. There was paresis of the legs, he could only walk with two sticks, and could not raise the legs off the bed, nor when seated with his feet on the ground the toes from the floor. There was no wasting of muscles, nor alteration in electrical reactions. Each leg as high as the knee was the seat of "stocking" anæsthesia, of which the upper border was sharply defined. Loss to light touch and hot and cold was complete; to pain (pin-prick) partial only.

In some cases where hysterical monoplegia has lasted for a considerable time there may be definite wasting of the muscles of the limb. This wasting is of slight degree and affects the muscles of the whole limb, as in disuse. The electrical reactions are retained. Thus a man who had a superficial wound above the fold of the elbow which could not have injured the nerves or any deep structures, complained of paralysis of the forearm and hand. There was distinct but slight wasting of all the paralysed muscles. The wrist-jerks were feeble or absent, elbow-jerks unaffected. In this case there were no sensory disorders. He made a good recovery.

In dealing with all these cases, the essential point is for the observer to be absolutely confident of the diagnosis; failure in treatment may result from some doubt in his mind as to the correct diagnosis, and hence hesitancy in carrying out appropriate measures of treatment, with consequent failure to gain the patient's confidence.

Another form of hysterical disorder which has come under observation is the common type of aphonia succeeding a laryngeal catarrh, which has shown the same tendency to relapse and the same difficulty of permanent cure that is met with in civil practice.

We have had a number of cases of complete dumbness with or without deafness from shell-shock. In most cases the cause was the shock of a shell explosion, with or without burial, sometimes producing loss of consciousness for varying periods, sometimes not, but in either case leaving the patient in a dull, dazed or stuporous state, from which he emerged to find himself dumb and often deaf as well. When the result was both deafness and dumbness, in most cases hearing was recovered before speech, but in two speech before hearing. Recovery took place in some quite suddenly, sometimes at a concert, or under some excitement, but in others gradually, with the recovery of a few words first. In these the recovery was attended by a marked stutter. Recovery was aided by demonstrating to them the physiological movements of the lips and tongue in forming labials and linguals and getting them to imitate the movements, first of the " voiceless consonants." The patients were generally able to read and write, to understand writing, and to a limited extent use gestures. In the modes of recovery the cases corresponded to what is seen in hysterical dumbness, but in some other respects several of them differed from the behaviour of a patient with the classical type of this affection. For in this type the patient is eager to write what he cannot say, or tries to convey his meaning by elaborate and exaggerated gestures. He waits with pencil and paper ready for the doctor to reach his bed. Although some of the patients showed this eagerness, others were dull and apathetic, and apparently had little desire to attract notice or make themselves understood.

Similarly in a case of deafness from shock without lesions of the ears the patient did not make the efforts to hear that a deaf person does, *i.e.* he did not use the gestures or attitudes of the deaf, and had not the expression of one trying hard to hear.

Hysterical vomiting has occasionally occurred, either as an isolated symptom, or associated with other symptoms of shock. This affection is apt to be refractory to treatment. In two obstinate cases no improvement was obtained until the patient was strictly isolated. Treatment by neglect of the symptom or by putting the patient at once on to a full diet whilst isolated, which is often a most successful plan in civil practice, here failed. The patients were cured by keeping them strictly to milk until vomiting had ceased for some time, and then gradually getting them back to a full diet. It was difficult in a few of these forms of hysterical disorder not to think that the patient has little desire to get well, and it takes time and some insistence before he can be got into a more healthy frame of mind.

Except in one instance, I have seen no case of hysterical convulsions, which is perhaps contrary to what one would have expected. This patient had severe fits (hystero-epilepsy), occurring in series. He was twenty years of age, and had never suffered from epilepsy. He had a slight wound, and fell back into the trench from a height of six feet, striking the back of his head. There was no mark from this injury. He was drowsy and mentally dull on admission, and a week later began to suffer from fits. They followed one another at short intervals, in series lasting from one to two hours. In the fits the arms were first raised and extended in clonic spasm, if held he resisted violently, he then turned on to his right side with rigid extension of legs and back in opisthotonus. There were irregular movements of the eyeballs, and an interesting feature was well-marked Hippus. Though the tongue was protruded, it was never bitten. Complete loss of consciousness were doubtful. He was morose and sullen between the attacks, when he had paresis with ataxy of movement of the left leg, which was anæsthetic up to the knee. There was also "glove" anæsthesia of the right forearm and hand. The visual fields were concentrically contracted. These fits recurred with intervals of a day or two for about a fortnight, he was then strictly isolated in a small ward with an observation window and his bed made up on the floor. For a week he had occasional very slight attacks, generally when the nurse came into the ward. No notice was taken of them, and in a fortnight they had altogether ceased. The paresis of the leg and the anæsthesia cleared up without any treatment. He returned to the general ward, and remained there about three weeks, to make sure no relapse would occur. At first he was dull and listless, but later cheered up and made himself useful in the ward. He was discharged well. Probably he was not quite up to the normal intelligence.

In the above cases which show various forms of hysteria in some there is freedom from symptoms of nervous shock, either because of the conditions under which the affection originated, or because such symptoms, though present at first, had passed off before the patient came under notice; but in others the hysterical features were accompanied with more or fewer evidences of that state of general nervous shock which was present at some time in all the cases now to be considered. We are here dealing with neuroses which present symptoms or groups of symptoms not familiar in civil practice before the war. With regard to these war neuroses, I refer again to what I stated at first, that their exact pathology is doubtful, and their nature not always the same, not due to the same cause, and that it is possible that some of them are due to direct mechanical injury to the brain, cord, or nerves from the violent concussion of high explosives, yet without signs of external injury. As an extreme instance of what may occur in this way, a patient under my care has all the signs of a total transverse lesion of the cord in the lower dorsal region, including a huge sacral bedsore, from the explosion of a shell in his immediate neighbourhood, and yet there is no damage to the spinal column nor any external mark of injury.

Apart from such a case, where the evidence of severe structural damage is conclusive, I may also reiterate that the absence of the clinical signs of structural change in the central nervous system does not necessarily prove that there are none, especially of the nature of minute but numerous lesions. In this connection the lectures of Dr. Mott,[1] especially with regard to the effect of CO poisoning, deserve careful study.

For the reasons already given, I have taken a clinical basis for the following observations, namely the absence by the ordinary clinical methods of investigation of any signs of a gross or structural lesion of the nervous system.

The causes of the war neuroses are manifold, and comprise the effects of anxiety, overstrain, of want of sleep, of wounds of varying severity, of the concussion from high explosives, of the perpetual noises which probably have a cumulative action upon the nervous system, of the horrors of the scenes through which the patient has passed, and in some cases, and very naturally, of fear. But the most potent factors in causation of nervous shock are the concussions caused by high explosives and burial in the debris produced by a bursting shell. These were the especial causes in the most severe cases under my care, and the longer the patient was buried before being dug out the greater the effect. I am in agreement with those who have found that in the majority of these patients, when the family history is inquired into, there is evidence of some neurosis or nervous disease in the patient's family.

Sometimes the patients have previously stood the strain well, but began to show the effects of nervous shock or fright

[1] *Lancet*, 1916, vol. i. pp. 441 *seq.*

when under fire after an attack of dysentery, of influenza, or some similar debilitating disease.

In all cases, whatever the variety of the predominant features, there are certain common symptoms, varying in degree, and not all present in the same case, of which the most important are exhaustion or prostration, both bodily and mental, with consequent lack of interest in surroundings, even in the progress of their own illness, and sometimes even an absence of desire to get better. For a varying time, often a long one, the patients are incapable of making any effort, and exertion of any kind entails speedy fatigue. Mental lethargy may be marked and persistent. There is often wasting or disturbance of nutrition, with or without anorexia. Neither glycosuria nor albuminuria are features of the condition.

The mental state in the early stages of the illness is one of depression, with loss of self-confidence. I have not met with hallucinations or delusions, except in patients suffering from a definite psychosis. The depression is often associated with fears of permanent paralysis or ill-health. Even when this is not mentioned, it is often at the back of the patient's mind, and if satisfactory assurance can be given on this point, there is often a speedy improvement in the depression. Tremors of the limbs may be a source of worry. The patients are extraordinarily sensitive to noises, and sudden, unexpected noises may upset them for a day. Often a sudden noise produces a sense of spasm of the chest and difficulty in breathing ; possibly this, in cases where CO poisoning has played a part, may be an unconscious revival of their first symptoms. Hence at first they prefer to be, and get on better, alone in a small ward. A sign that the first stage of irritability with depression is passing off, is a request to be moved into the general ward. Exceptionally they are afraid to be alone, and will rather put up with the discomfort from the noise inseparable from a large ward than be by themselves.

Concentration is slow, thought painful; when asked to carry out three simple orders this will be done very slowly in the early stages of illness, and in the majority of cases incorrectly, obviously involving a painful effort.

Memory for events following the injury is imperfect, and immediate memory often very poor, though rarely so bad that the patient cannot remember if he has had his breakfast or dinner. From this cause, and from difficulty of concentrating the attention, the patients at first rarely read, for they find they cannot follow a connected narration of events or a story. Another sign of improvement is when the patient voluntarily begins to read. In the milder cases memory for remote events before the war is not affected, but in the more severe this also may be bad, even for incidents of boyhood. No doubt difficulty in reading or carrying on any occupation needing good vision is often largely due to easy fatigue of the muscles of accommodation, and blurring of vision from this cause is a common symptom at first. The mechanical act of writing is often good, but at the same time writing a letter may be a very arduous task; the difficulty here is in the construction of sentences.

Affections of the special senses—amaurosis, deafness, loss of smell and taste—are common soon after the accident, but seldom persist long. A moderate degree of deafness is the most persistent.

Nystagmoid movements of the globes are not infrequent, definite nystagmus I have only seen in one case. In view of the extreme proneness to fatigue, a medical examination has at first to be carried out with caution, as if unduly prolonged, it may throw the patient back for days. Hence it is rarely possible, and certainly undesirable, to attempt a complete examination at one sitting. The same applies to exercises when these are begun : if unduly pushed at first, the patient may be upset for two or three days, and lose sleep and appetite. To many the nights are a source of dread. More or less insomnia is at first the rule, and sleep is disturbed by terrifying dreams. The most common dream is a sense of falling through space. Other dreams repeat incidents of the fighting ; the patient is buried again in the trench, or is in a hole and cannot get out, or is being attacked by Turks, or grasped by the enemy, and cannot get away. From such a dream the patient wakes sweating profusely and in a general tremor ; sleep is often gone for the rest of the night, which is filled with visions of dreadful experiences and sights seen in the trenches, and as a result, if the patient is suffering from tremors of the limbs, these are increased during the next day.

Less commonly the patient wakes suddenly in a similar agitation, but cannot recall the actual dream. Occasionally there are day dreams, or states in which a phantasmagoria of incidents of the fighting pass before his eyes. With regard to tremors, there is often a fine general tremor of the limbs, occasionally present during rest, more often conspicuous on movement. In one case the tremor exactly resembled that of disseminated sclerosis, but as a rule it is a fine tremor. In a few cases the head trembled whilst the limbs were steady. Further, in two or three instances in which there was a marked loss of the muscular sense, the tremor resembled that of Friedreich's disease, the hand making swooping movements before attaining its destination.

Of course tremor, especially of the head or legs, is one effect of the emotion of fear which is at work in some of the cases. I think that excessive tobacco smoking is in man a contributing cause of tremor.

Such being a short summary of the more striking general symptoms, it should now be said that these are associated in many cases with paralysis of some part of the body. In some, from the first, standing out from the general disorders, there is a more defined loss of power either hemiplegic or paraplegic in distribution. When a localised paralysis starts from the time of the injury, it is more frequently hemiplegic. But more commonly there is at first more or less general loss of power of all the muscles of limbs and trunk, and later, often after some weeks, as the signs of general shock begin to pass off, those of a more localised affection declare themselves, and this is especially a paraplegia. The cranial nerves, other than the oculars in the forms above mentioned, invariably escape.

The chief features can be best illustrated by an example of a severe case in which all the limbs were affected at first, the upper extremities slowly recovered, and paraplegia remained. Sensory disorders were especially prominent. The patient, a man aged forty, received an injury to the head sufficient to cause a scalp wound, but no more serious damage; if there was any loss of consciousness it was very brief. On admission, three months after the injury, he was fat and looked well, but could not stand or walk, and his hands and arms were very feeble. He complained of headache, slept badly, and had a poor appetite. Except for occasionally glancing at the paper, he was content to lie all day doing nothing, in a state of mental inertia. Reading and writing were difficult because any effort readily produced extreme fatigue; moreover, his memory was bad, both for remote events and those of the day, *e.g.* he could not remember where he went to school, and was not always sure whether he had had his dinner. All answers to questions were made very slowly and after some interval.

He could feed himself slowly, carry out a few movements of arms and hands, and just raise his feet from the bed. The muscles were not wasted; when passively moved there was a sort of plastic state, not true rigidity, and a tonic spasm was sometimes induced thereby. A brief repitition of the few voluntary movements possible resulted in the muscles passing into a flaccid condition. Arm movements were attended with the "swooping" tremor above described. Superficial reflexes were normal, deep exaggerated.

On the sensory side concentric contraction of the visual fields was easily induced by testing them. There was all over the body a certain dulness to perception of a light touch or pin-prick, attributed to mental dulness. With his eyes shut he did not recognise objects placed in his hands, and had absolutely no idea of the position of any of his limbs, nor whether they had been moved, except that he recognised vaguely movement of the large joints through an angle of 90 degrees. Moreover, if I held up his left hand and asked him to touch his left forefinger with his right forefinger, he would completely fail to do so; but if he touched my hand instead, would keep his finger on it, and rest satisfied that he had correctly done what I had asked. Sometimes he was satisfied by merely making a movement of the finger in any direction. If I then moved his hand away, he was completely lost as to its position. There was one interesting point, if he had once seen the position of a limb and it was not moved, he remembered its position, and could touch it even after a long interval.

Localisation was very imperfect for instance; on both hands it was from wo inches to four inches out, generally in a proximal direction on long axis of the limb, he rarely failed altogether, but was always very slow.

After two months he was somewhat less dull mentally, and took more interest in his surroundings, but was still inert His memory had improved, he read more, and was successfully making a rug. He had now fair use of his arms and hands. The legs had lost the little movement possible on admission, they were more anæsthetic to touch and pain —he had no sense of their position or movement, and there was distinct cataleptic rigidity, *i.e.* when placed in any position they remained rigidly fixed in this position for some time. He could sit up in bed. Reflexes were in the same condition as before. State of nutrition of the muscles good, and electrical reactions normal throughout. He appeared to be capable of making very little effort towards recovery, and except for the recovery of the arms, the result of various kinds of treatment has so far been unsatisfactory.

In the cases in which hemiplegia was the predominant symptom there was as a rule a period of unconsciousness after the injury, whether due to the explosion of a shell or to being buried in the debris of a trench or to shell or bomb wounds on the trunk or limbs. The hemiplegia, sometimes at first complete, was subsequently a more or less severe heim-paresis. The deep reflexes were exaggerated on the affected side. The paresis was frequently accompanied by some ataxy or inco-ordination of movement.

Sensory symptoms of a certain kind were marked. Common sensation was as a rule unaffected, and the hemi-anæsthesia of hysterical hemiplegia, an important point in discrimination, was not found. In one case there was loss of light touch over the hand and forearm, and also asynergia and apraxia of this limb. With eyes shut movement was inco-ordinate, he could not recognise common objects placed in the hand, nor put them to their proper use, nor discriminate between weights, and he had no knowledge of the position of his hand.

In the other cases objects were recognised, though generally slowly; but there was defect or loss of localisation of touch, defective discrimination of two points at the normal distance, or total failure to discriminate them, loss of power to appreciate different weights, and of the position and of degree of passive movements of the affected limb. For the latter I used Dr. Head's method of testing, and I gladly here express my indebtedness to his writings and demonstrations on these features.

Localisation was generally, though by no means invariably, proximal along axis of limb to the spot touched. Sense of movement was most imperfect at the periphery, thus it might be completely lost for the fingers, very imperfect at the wrist, and fair or good at the elbow, and good at the shoulder. The same applies to the lower extremity. In one case there was the very interesting symptom that the patient, if he put down an object he had been holding in the affected hand, entirely forgot where he had placed it, whilst when using the other hand he had the normal memory of what he had done.

In all cases electrical reactions were normal : the muscles on the affected side were flabby without sign of rigidity at any period of the case.

In one case on the hemiplegia side alone there developed gradually a very marked intention termor, which was practically indistinguishable from that of disseminated sclerosis, and which persisted so long as he was under observation. Other evidences of disseminated sclerosis were, however, absent.

The results in these hemi-paretic cases were good. Treatment by rest, good feeding, massage, passive movements and exercises resulted in restoration to health. The leg in all cases recovered before the arm.

More common than hemiplegia or hemi-paresis as the residual affection, when the general symptoms of nervous shock are in process of subsiding, is some form of paraplegia ; a monoplegia I have not come across.

These paraplegias are of all grades of severity : two examples will serve to illustrate the milder forms.

A man was buried twice and concussed by shell fire: he became unconscious and remembered no more until four days later, when he recovered to find himself partially blind and deaf, with loss of smell and taste and paraplegic. On admission fourteen days later he had recovered the use of his special senses and the general symptoms of shock were passing off, but he could not walk, and could only stand with support. He was much troubled by fears of paralysis. He had slight defect of sense of position and of movement of legs, but no other sensory changes. All reflexes normal. When he began to try to walk he had an extraordinary gait; the legs were held absolutely rigid and violently jerked out, whilst his head and trunk were thrown so far back that he looked as if he would fall on the back of his head. The nature of his trouble was explained to him, and being an intelligent man he steadily persevered, aided by massage and passive movements, and in three weeks had completely recovered. He said that "he had fought hard to recover."

In another patient who was blown up by a mine and was unconscious for three days, the signs of nervous shock were still severe on admission and he was kept in bed in a ward by himself for three weeks, until they gradually improved; at the end of this time, when he was allowed to get up, it was found that he could not walk, and could only stand by holding on to something. There was a tremor of the legs, and he said that he felt very uncertain as to their position, though all that could be found on examination was some defect in the senses of position and of movement of the right foot only. At first when he tried to walk his feet seemed to cling to the floor, so that he scraped them along it, whilst at the same time the legs were agitated by a coarse tremor or clonic spasm of the muscles and he brought each leg forward with a violent muscular effort, an appearance further borne out by the increased rapidity of pulse and respiration. He recovered more slowly, and at the end of a month walked normally, though if he went far the tremor of the legs returned.

In these milder cases, with their individual peculiarities of gait, there is nothing that is not seen occasionally in ordinary practice, but in the severer cases of paraplegia there are more unusual features. In these severe cases there is a good deal of individual difference. In some there are marked disturbances of the deep sensibility and of localisation of touch without muscular wasting or evident loss of muscular tone. This type has already been sufficiently described above. In others the paraplegia was also complete, long lasting, without or with slight sensory disorder, but with flabby, flaccid and wasted muscles, the wasting of the leg muscles being general. In this type of case, which was to me at any rate a new one, there was a varying degree of tenderness of the dorsi-lumbar spine with stiffness on movement. The muscles were often tender. The electrical reactions were normal to Faradism and to the Condenser, but increased to Galvanism without qualitative change at the two poles; continued Faradism did not quickly exhaust the muscles.

The deep reflexes were exaggerated, true ankle-clonus was not found, Oppenheim's reflex occasionally, the plantar was never a true extensor, but sometimes there was a quick extension of the whole foot.

In one case there was in addition to general also more pronouced local wasting of one Quadriceps Extensor; this muscle showed decreased response to both Faradism and Galvanism and KCC = ACC.

The patients could move their legs to a limited extent when lying in bed, but the repetition of such slight voluntary movements as were possible quickly tired them out; further, these movements showed very peculiar features. To produce a slight movement gave the appearance of a man making an extraordinary effort, as of one lifting a heavy weight, all the muscles concerned in the movement, both prime movers and antagonists entered into (1) tonic spasm lasting some seconds, succeeded by (2) slow clonic contractions of the same muscles, which slowly subsided as (3) the desired movement was finally carried out. These phenomena were repeated each time the movement was made.

In addition there was sometimes also a peculiar and allied modification of the tendon-phenomenon. If the patellar tendon was struck two or three times in succession there followed, first, a rather slow jerk, and then a sustained spasm of the Quadriceps, in which the muscle remained firmly contracted for from 15 to 30 seconds, and then slowly relaxed.

When the patients tried to stand or walk their legs doubled up or slipped from under them. The patient may know perfectly the position of his limbs, and in bed both deep sensibility and common sensation seemed to be unaffected, and yet had no control over them when out of it. The sphincters were unaffected.

Three of the patients recovered completely, with the others little progress has so far been made. The patients who recovered after preliminary treatment by massage, electricity and passive movements, under which the muscles gained in size and tone, had to be taught to walk step by step, and did not improve much until it was realised that they must be shown how to carry out each detail of the movements of walking, then they made fairly rapid progress. It was not so much that they were devoid of the sense of position or movement of the legs, but that they seemed to have lost the automatic or subconscious representative mechanisms of the movements of walking, which had to be built up again. One movement, extension of toes with dorsiflexion of foot, seemed especially difficult for them.

Looking at the cases as a whole, the conclusion seems inevitable that whatever the exact nature of the pathological changes, they must be widely distributed through the nervous system. In accounting for the symptoms, we must not leave out the mental state at one end of the scale, nor the failure in muscular nutrition at the other. The disturbance affects the highest cortical levels, the middle levels with the subconscious mechanisms for everyday activities, the motor centres in the cord with their issue in the final common path, and the muscles themselves, and often also the afferent paths and the receptive apparatus for localisation and the components of deep sensibility. The variation of symptoms in individual cases will depend upon what paths, centre or centres the incidence chiefly falls.

Where disorders of deep sensibility are prominent this is probably sufficient to account for the disorder of movement. The dull mental state of the early stages, suggest that cortical voluntary impulses are then defective. In other patients the condition of the muscular apparatus and presumably of the spinal motor cells on which their nutrition depends, would alone be sufficient to cause paralysis, even if the higher nervous system were intact.

Though one must be guarded in drawing conclusions from appearance only, the impression generally conveyed is that of a block in the passage of nervous impulses from one neuron to another. Especially is this so in those cases in which the mind seems to have fully recovered, and the muscles of the legs are strong and well nourished, and yet the patient has no power to move them. It looks here as if the resistance of the synapses has been so greatly increased that impulses could no longer pass. It has been suggested, I think by Dr. Mott, that one effect of the concussion from high explosives may be to cause a retraction of the terminals of neurons, and so leave a space over which impulses cannot pass. It is possible, however, that the difficulty may be due, not to separation in space, but to alteration in the constitution of the terminal ramifications of the axones and of the dendrites.

The peculiar disorder of voluntary movement described above shows, if my observations are correct, disturbance of the reciprocal innervation through the final common path of the muscles concerned in the movement Instead of the normal process of a maximum innervation of the prime movers and a simultaneous inhibition of their antagonists, or at most as in some movements a moderated contraction of antagonists for the purpose of ensuring steadiness of a movement, in this disorder both agonists and antagonists are strongly innervated together, the result being not the desired movement, but a spasm of the limb in the position of the strongest set of muscles.

I would further refer this disorder of voluntary movement to an overaction of the cerebellum, or perhaps rather to the want of counteraction of the cerebellum, owing to the impulses from the cerebrum being in abeyance.

Briefly stated, the argument in favour of this view of unopposed or exalted cerebellar action is based, (1) on the dual structure of muscles, for according to Boeck's morphological researches one component, a sarcoplasmatic mass, is innervated by a sympathetic motor cell, and the other, the striped apparatus, by an anterior horn cell; (2) on Langelaan's observations on muscle tonus, in which, in experiments on tendon-reflex phenomena, a tonic tetanus is shown to precede clonus, and to be due to exalted irritability of the proprio-ceptive arc, and clonus itself to be a series of twitches (due to the anterior horn and striped apparatus component of muscle), superimposed upon a tonic tetanus, (due to the sympathetic component) ; and (3) on Sherrington's view that the cerebellum is the main ganglion of the proprioceptive system[1].

Finally, I may say, and it is perhaps contrary to expectation, that cases of conscious simulation of nervous disorders have been conspicuous by their absence, and that the influence of fear is not so great nor so lasting as one would have anticipated, for all men are not constitutionally brave, and the conditions of modern warfare are such as to daunt the stoutest heart. That, however, in a few the fear of a return to the front does retard recovery is certain, and in the case of a man for whom there is no possible prospect of returning to active service it is well to tell him so.

---

### NOTES ON THE ENTERIC GROUP OF DISEASES AS SEEN IN AN ISOLATION HOSPITAL IN FRANCE.

BY

J. M. Fortescue-Brickdale, M.A., M.D., M.R.C.P.,

Captain R.A.M.C. (T.).

---

In this paper I propose describing first the general arrangements of the hospital to which I was attached and the measures taken to isolate patients and prevent the spread of infection, and in the second part to give my own impressions as to the clinical features of the enteric group of cases, more especially with reference to Paratyphoid B.

The hospital consisted primarily of eight huts, each

[1] For these points I am indebted to Langelaan's valuable paper on "Muscle Tonus—Brain," vol. xxxviii. part iii. p. 235. Although his experiments quoted refer primarily to tendon-reflex phenomena, and the disorder I describe is one of voluntary movement, yet they seem to me comparable, especially in view of the peculiar modification of the knee-jerk, which sometimes was present in the same patient.

